# Overall survival in the SIMPLIFY-1 and SIMPLIFY-2 phase 3 trials of momelotinib in patients with myelofibrosis

**DOI:** 10.1038/s41375-022-01637-7

**Published:** 2022-07-22

**Authors:** Ruben Mesa, Claire Harrison, Stephen T. Oh, Aaron T. Gerds, Vikas Gupta, John Catalano, Francisco Cervantes, Timothy Devos, Marek Hus, Jean-Jacques Kiladjian, Ewa Lech-Maranda, Donal McLornan, Alessandro M. Vannucchi, Uwe Platzbecker, Mei Huang, Bryan Strouse, Barbara Klencke, Srdan Verstovsek

**Affiliations:** 1 UT Health San Antonio Cancer Center, San Antonio, TX USA; 2 Guy’s and St Thomas’ NHS Foundation Trust, London, United Kingdom; 3 Washington University School of Medicine, St. Louis, MO USA; 4 Cleveland Clinic Taussig Cancer Institute, Cleveland, OH USA; 5 Princess Margaret Cancer Centre, Toronto, ON Canada; 6 Monash University & Frankston Hospital, Frankston, Australia; 7 Hospital Clinic, IDIBAPS, University of Barcelona, Barcelona, Spain; 8 Department of Hematology, University Hospitals Leuven and Department of Microbiology and Immunology, Laboratory of Molecular Immunology (Rega Institute), KU Leuven, Leuven, Belgium; 9 Medical University of Lublin, Lublin, Poland; 10 Université de Paris, AP-HP, Hôpital Saint-Louis, Centre d’Investigations Cliniques, INSERM, CIC1427 Paris, France; 11 Institute of Hematology and Transfusion Medicine, Warsaw, Poland; 12 University of Florence and AOU Careggi, Florence, Italy; 13 Leipzig University Hospital, Leipzig, Germany; 14 Sierra Oncology Inc, San Mateo, CA USA; 15 The University of Texas MD Anderson Cancer Center, Houston, TX USA

**Keywords:** Targeted therapies, Myeloproliferative disease

## Abstract

Janus kinase inhibitors (JAKi) approved for myelofibrosis provide spleen and symptom improvements but do not address anemia, a negative prognostic factor. Momelotinib, an inhibitor of ACVR1/ALK2, JAK1 and JAK2, demonstrated activity against anemia, symptoms, and splenomegaly in the phase 3 SIMPLIFY trials. Here, we report mature overall survival (OS) and leukemia-free survival (LFS) from both studies, and retrospective analyses of baseline characteristics and efficacy endpoints for OS associations. Survival distributions were similar between JAKi-naïve patients randomized to momelotinib, or ruxolitinib then momelotinib, in SIMPLIFY-1 (OS HR = 1.02 [0.73, 1.43]; LFS HR = 1.08 [0.78, 1.50]). Two-year OS and LFS were 81.6% and 80.7% with momelotinib and 80.6% and 79.3% with ruxolitinib then momelotinib. In ruxolitinib-exposed patients in SIMPLIFY-2, two-year OS and LFS were 65.8% and 64.2% with momelotinib and 61.2% and 59.7% with best available therapy then momelotinib (OS HR = 0.98 [0.59, 1.62]; LFS HR = 0.97 [0.59, 1.60]). Baseline transfusion independence (TI) was associated with improved survival in both studies (SIMPLIFY-1 HR = 0.474, *p* = 0.0001; SIMPLIFY-2 HR = 0.226, *p* = 0.0005). Week 24 TI response in JAKi-naïve, momelotinib-randomized patients was associated with improved OS in univariate (HR = 0.323; *p* < 0.0001) and multivariate (HR = 0.311; *p* < 0.0001) analyses. These findings underscore the importance of achieving or maintaining TI in myelofibrosis, supporting the clinical relevance of momelotinib’s pro-erythropoietic mechanism of action, and potentially informing treatment decision-making.

## Introduction

Myelofibrosis (MF) is a chronic, progressive, Philadelphia-negative myeloproliferative neoplasm characterized by dysregulated JAK-STAT signaling and aberrant inflammatory cytokine production, with features of bone marrow fibrosis, anemia, splenomegaly, burdensome symptoms (i.e., fatigue, cachexia, fever, night sweats), tendency to leukemic transformation, and shortened survival [1]. MF may present de novo (primary MF, or PMF) or secondary to polycythemia vera (post-PV MF) or essential thrombocythemia (post-ET MF). Due to the heterogeneity of clinicopathological features of MF, validated prognostic scoring systems facilitate patient risk stratification and inform clinical decision-making. Among MF clinical indicators, two of the foremost negative prognostic factors are anemia and transfusion dependency, each of which are independently inversely correlated with overall survival (OS) and quality of life [2–4]. The Dynamic International Prognostic Scoring System (DIPSS) places a two-fold higher weight in risk score on a hemoglobin (Hgb) level <10 g/dL, and the DIPSS-plus model incorporates transfusion dependency as an additional prognostically adverse factor that further elevates risk [3, 4]. Patients with mild, moderate, and severe anemia experience shortened median survival of 4.9 years, 3.4 years, and 2.1 years, respectively, and the risk of death is 1.5-fold higher in severely anemic versus moderately anemic patients [5]. Similarly, when patients are stratified by risk category, OS is markedly worse for intermediate-2 and high-risk patients at 4 years and 2.25 years, respectively, compared to a median OS of ~6 years among all patients with MF [2, 6]. Because approximately 60% of patients with MF are anemic within one year of diagnosis, and nearly all become dependent on red blood cell transfusions over time [7, 8], anemia remains one of the most critical disease facets to address.

Allogeneic hematopoietic cell transplantation (HCT) is the only curative therapy for MF and has shown significant improvements in OS [9]. However, HCT is restricted to a limited subset of non-elderly (< 70 years of age) patients with viable donors and good performance status, as the procedure is associated with high morbidity and mortality particularly in older adults [10]. For many patients who are ineligible for HCT, the current standard of care entails treatment with one of the currently approved JAK inhibitors (JAKi).

Ruxolitinib (RUX), the first FDA-approved and primary JAKi used to treat MF over the past decade, has demonstrated spleen and symptom improvements in patients with MF but is myelosuppressive and associated with dose-dependent anemia [11, 12]. Long-term pooled analyses from the COMFORT studies showed a prolonged median OS of 5.3 years with RUX compared to 3.8 years with placebo or best available therapy (BAT) in patients with intermediate-2 or high-risk MF (HR 0.70; *p* = 0.0065) [13]. OS was not significantly different between RUX-treated patients who were transfusion independent and not transfusion independent at week 24 [13], whereas a significant prognostic improvement was demonstrated in RUX-treated patients who experienced spleen volume reduction at week 24 compared to those who did not [14]. In an observational study of RUX-treated patients with MF, red blood cell transfusion need at baseline, 3 months, and 6 months of treatment was negatively correlated with OS [15]. Further, a real-world study of intermediate- to high-risk MF patients demonstrated that OS was significantly improved in the five years following RUX approval compared with the two years prior; notably, in the RUX post-approval time frame, OS was greater for those who received RUX versus those who did not [16].

Despite the advances achieved in MF due to RUX, cytopenia remains a problem. An exploratory analysis from the COMFORT studies found that anemia worsened in 69% of patients with baseline anemia following treatment with RUX, and 61% of patients who did not have baseline anemia experienced on-treatment anemia [17]. Often, patients receive attenuated doses of RUX due to anemia or reduced platelet counts, which may impact treatment efficacy, and some never initiate JAKi therapy to avoid worsening their disease-related bone marrow impairment. Even among patients who initially benefit from RUX, eventual discontinuation may be necessary due to intolerance, disease progression, or sub-optimal response often corresponding with cytopenia-related dose reductions. While clinical trial data show that half of patients discontinue RUX within 3 years [11, 12], real-world evidence suggests approximately 40–70% of MF patients discontinue RUX during the first year of treatment with anemia being the leading cause [18–20]. Because patient survival following RUX discontinuation is generally poor [21, 22], there is a substantial unmet medical need for safe and efficacious therapies for MF patients presenting with anemia and in those previously treated with an approved JAKi.

Momelotinib (MMB), the first JAK1 and JAK2 inhibitor to also inhibit activin A receptor type 1/activin receptor-like kinase-2 (ACVR1/ALK2), has been investigated in >1000 MF patients, including in the completed phase 3 studies in JAK inhibitor-naïve (SIMPLIFY-1) and previously RUX-exposed (SIMPLIFY-2) patients [23, 24]. MMB has demonstrated clinical activity against anemia, symptoms, and splenomegaly, and confers substantial anemia benefits including conversion to and maintenance of durable transfusion independence, reductions in transfusion burden, elevated Hgb levels, and fewer adverse events of anemia in phase 3 clinical trials [23–25]. Preclinical and clinical translational studies have demonstrated that MMB’s ability to improve anemia and transfusion dependency is linked to suppression of ACVR1/ALK2-mediated hepcidin production, which leads to increased serum iron availability and stimulation of erythropoiesis [26, 27]. Importantly, elevated hepcidin is significantly associated with shortened OS in patients with MF [28]. However, the impact of MMB treatment and response on OS and leukemia-free survival (LFS) has not yet been reported in the literature.

Here, we present mature analyses of OS and LFS observed with extended MMB treatment in the SIMPLIFY-1 and SIMPLIFY-2 study populations. Associations between baseline characteristics, as well as week 24 clinical responses, and OS were also examined.

## Methods

### Clinical study design

The details of SIMPLIFY-1 (NCT01969838) and SIMPLIFY-2 (NCT02101268) study designs have been previously published [23, 24]. SIMPLIFY-1 was a randomized, double-blind, phase 3 study conducted in JAK inhibitor-naïve intermediate- and high-risk patients with PMF, post-PV MF, or post-ET MF (*n* = 432) designed to test the non-inferiority of outcomes for subjects randomized 1:1 to receive MMB or RUX over a 24-week treatment period, at which time patients originally randomized to MMB were able to continue receiving the compound for an extended treatment period, while RUX-randomized patients were eligible to cross over to MMB therapy. In total, 430 subjects received at least one dose of study treatment, and 197/216 (91.2%) of RUX-randomized patients crossed over to MMB treatment at week 24. SIMPLIFY-2 was a 2:1 randomized, multinational, open-label phase 3 study testing the superiority of MMB compared to best available therapy (BAT, which included RUX in 88.5% of patients) in patients with PMF, post-PV MF, or post-ET MF who experienced hematologic toxicity when previously treated with RUX (*n* = 156). All 156 subjects received study treatment, and 40/52 (76.9%) of BAT/RUX-randomized patients crossed over to MMB treatment at week 24. In both trials, the primary endpoint was spleen volume reduction (SVR) ≥ 35% from baseline at 24 weeks. Secondary endpoints included total symptom score (TSS) response rate and red blood cell transfusion independence (TI) rate at 24 weeks and overall and leukemia-free survival (OS and LFS).

Patients whose disease did not progress and who tolerated MMB treatment while enrolled in SIMPLIFY-1 or SIMPLIFY-2 were eligible to enroll in an ongoing open-label, extended access protocol (NCT03441113) at the completion of these phase 3 studies. Survival data captured during this extension protocol were included in the analyses presented herein.

### Clinical response definitions

TI responders were defined as patients who did not receive a red blood cell transfusion and whose Hgb remained ≥8 g/dL within the 12 weeks immediately prior to week 24. SVR responders were defined as patients who experienced ≥35% SVR at week 24 compared to baseline. Symptom responders were defined as patients who experienced ≥50% reduction in TSS as measured by the Myeloproliferative Neoplasm Symptom Assessment Form (MPN-SAF) at week 24 compared to baseline.

### Statistical analyses

OS and LFS were analyzed for the enrolled populations from SIMPLIFY-1 and SIMPLIFY-2, inclusive of data for those who continued MMB treatment beyond study through the extended access protocol, using Kaplan-Meier analyses and compared between groups with stratified log-rank tests and proportional hazard Cox regression models stratified by randomization stratification factors. OS was calculated as time from first dose of study drug to death of any cause, and LFS was calculated as time from randomization to leukemic transformation or death from any cause, whichever earlier. Subjects who did not die or transform were censored at the last date known to be alive. No adjustments were made for the crossover of active control patients at week 24 in either study in the analyses because limited information is available for the minority of patients who did not cross over to MMB. There was no prespecified test for the difference between MMB and control arms for OS and LFS in the studies because it was recognized that the analyses would be confounded by the fact that all control patients were to cross over to MMB at week 24. Duration of follow up for OS and LFS was analyzed using the reverse Kaplan-Meier method.

Long-term safety data was analyzed from SIMPLIFY-1 and SIMPLIFY-2 studies and included patients who continued to receive MMB through the extended access protocol. Treatment emergent adverse events (TEAE) were documented for patients who received MMB, RUX or BAT/RUX in the 24-week randomization treatment periods and those who received MMB from crossover at week 24 throughout extended treatment and long-term follow up. The incidences of any grade TEAEs, and grade 3/4 TEAEs, by patient were calculated for each trial with a final data cut of September 2021.

The mean daily doses of administered MMB and RUX were calculated for patients on study each week over the 24-week randomization treatment periods in both SIMPLIFY-1 and SIMPLIFY-2. The mean daily doses of MMB were also similarly calculated for the subsequent week 24–48 extended treatment periods for both studies.

Univariate regression analysis was performed with the two treatment arms combined to explore associations between baseline characteristics and OS in SIMPLIFY-1 and SIMPLIFY-2 study populations. Baseline characteristics tested included TI status, TSS, Hgb level, spleen volume, platelet count, white blood cell (WBC) count, disease type and International Prognostic Scoring System (IPSS)/DIPPS risk category. A multivariate Cox regression model with stepwise selection was then used to select independent prognostic baseline factors.

To examine whether traditional MF endpoints of clinical efficacy (TI, SVR, and TSS response rates) at week 24 were predictive of improved OS in the SIMPLIFY studies, survival from week 24 was calculated for responders versus non-responders for individual clinical endpoints by treatment arm and analyzed using Kaplan-Meier analyses with log-rank tests to compare between groups, which included only those patients who were alive at week 24. Because the survival of RUX-randomized subjects in SIMPLIFY-1 and BAT/RUX-randomized patients in SIMPLIFY-2 were confounded by crossover to MMB at week 24, all responder analyses were performed separately for the MMB and control arms. Hazard ratios for MMB responders versus MMB non-responders were computed using proportional hazard Cox regression. Similarly, hazard ratios for responders versus non-responders for the control arm were also computed. Multivariate Cox regression analysis including all three response endpoints (week 24 TI, SVR, and TSS response rates) in the same model was performed to determine independence of associations between OS starting from week 24 and each response endpoint for MMB-randomized patients who were alive at week 24 in SIMPLIFY-1.

## Results

**Fig. 1:**
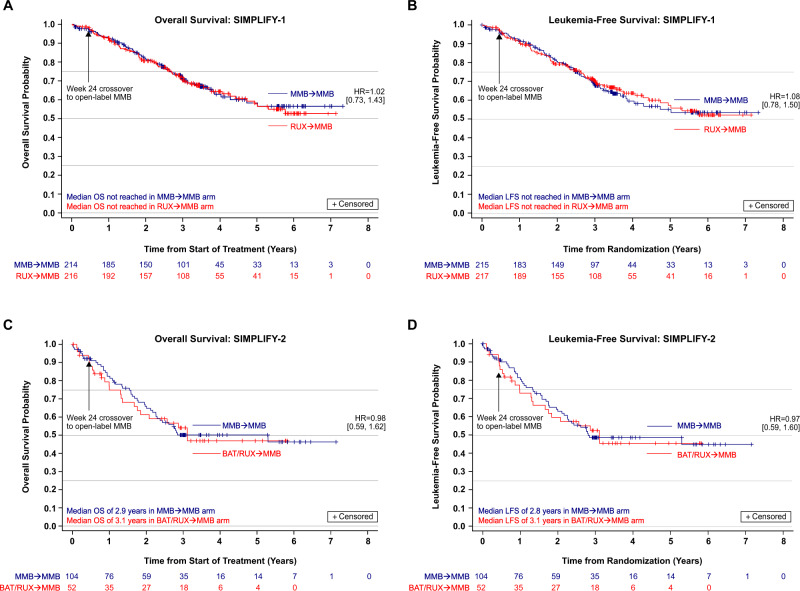
Overall survival (OS) and leukemia-free survival (LFS) of patients with MF in the phase 3 SIMPLIFY studies. **A**, **B** OS and LFS, respectively, in JAKi-naïve patients in SIMPLIFY-1; (**C**, **D**) OS and LFS, respectively, in JAKi-exposed patients in SIMPLIFY-2. From week 24, all patients who remained on therapy received open-label momelotinib (MMB). *RUX* ruxolitinib, *BAT* best available therapy, *HR* hazard ratio.

With a median follow-up of 3.43 years in the MMB arm and 3.47 years in the RUX arm of SIMPLIFY-1, 66 (30.8%) MMB-randomized patients and 73 (33.8%) RUX to MMB crossover patients died, and 12 (5.6%) MMB-randomized patients and 9 (4.2%) RUX to MMB crossover patients had leukemic progression. The distributions of OS and LFS were similar between RUX to MMB crossover patients and originally MMB-randomized patients (OS HR = 1.02 [95% CI: 0.73, 1.43], Fig. 1A; LFS HR = 1.08 [95% CI: 0.78, 1.50], Fig. 1B). The OS rates at 2, 4 and 6 years were 81.6%, 62.9% and 56.5% in the MMB arm and 80.6%, 64.4%, and 52.7% in the RUX to MMB crossover arm (Fig. 1A). The LFS rates at 2, 4 and 6 years were 80.7%, 59.7% and 53.6% in the MMB arm and 79.3%, 63.8%, and 52.2% in the RUX to MMB crossover arm (Fig. 1B).

With a median follow-up of 3.22 years in the BAT/RUX arm and 3.07 years in the MMB arm of SIMPLIFY-2, 47 (45.2%) MMB-randomized patients and 23 (44.2%) BAT/RUX to MMB crossover patients died, and 7 (6.7%) MMB-randomized patients and 1 (1.9%) BAT/RUX to MMB crossover patients had leukemic progression. Median OS from baseline was 3.1 [95% CI: 1.8, NE] years in BAT/RUX to MMB crossover patients and 2.9 [95% CI: 2.3, NE] years in originally MMB-randomized patients (HR = 0.98 [95% CI: 0.59, 1.62], Fig. 1C). The OS rate at 2 years was 65.8% in the MMB arm and 61.2% in the BAT/RUX to MMB crossover arm. Median LFS from baseline in SIMPLIFY-2 was 3.1 [95% CI: 1.7, NE] years in BAT/RUX to MMB crossover patients and 2.8 [95% CI: 2.3, NE] years in originally MMB-randomized patients (HR = 0.97 [95% CI: 0.59, 1.60, Fig. 1D). The LFS rate at 2 years was 64.2% in the MMB arm and 59.7% in the BAT/RUX to MMB crossover arm.

Safety of MMB was similar to that of RUX during the randomized treatment period of SIMPLIFY-1 with a few exceptions; all grade anemia and thrombocytopenia were less frequent while nausea was more common with MMB compared to RUX (Supplemental Table 1A). The incidence of grade 3/4 anemia was lower with MMB compared to RUX in both SIMPLIFY-1 (6.1% vs 22.7%, respectively) and SIMPLIFY-2 (13.5% vs 17.3%, respectively) (Supplemental Table 1). The median duration of MMB therapy was 17.7 months in SIMPLIFY-1 and 9.2 months in SIMPLIFY-2. Following closure of the SIMPLIFY studies, 118 patients who initiated MMB treatment through these trials continued to receive MMB through the extended access protocol, 88 of whom remained on MMB therapy for more than 5 years. No new safety signals or cumulative toxicity were observed during either randomized or extended MMB dosing (Supplemental Table 1). Furthermore, high MMB dose intensity was maintained throughout the 24-week study periods and beyond, whereas attenuated starting doses and progressive dose reductions of RUX were required due to induced or exacerbated myelosuppression (Supplemental Fig. 1).

In univariate analyses of baseline characteristics and OS in SIMPLIFY-1, TI status (*p* < 0.001), higher Hgb (*p* < 0.0001), and higher platelets (*p* = 0.0035) were each predictive of improved survival, whereas larger spleen volume (*p* = 0.0288), higher WBC (*p* = 0.0258), and IPSS high and intermediate (INT)-2 risk status (*p* < 0.0001) were predictive of shortened survival (Table 1). Multivariate analyses showed that IPSS risk status (HR [high versus INT-1]=4.293, *p* < 0.0001; HR [INT-2 versus INT-1]=2.759, *p* = 0.0044), WBC (HR [ ≥ 10 versus <10 × 10^9^/L] = 1.648, *p* = 0.0054) and TI status (HR [TI versus non-TI] = 0.474, *p* = 0.0001) at baseline were independent prognostic factors of OS after model selection (Table 2). Similar associations were observed in SIMPLIFY-2, where TI status (*p* = 0.0002), Hgb (*p* = 0.0003), spleen volume (*p* = 0.0006), WBC (*p* < 0.0001), and DIPSS risk status (*p* < 0.0001) were prognostic baseline characteristics in univariate analysis (Table 1). Multivariate analyses showed that in SIMPLIFY-2, TI status (HR [TI versus non-TI] = 0.226, *p* = 0.0005), Hgb (HR [8-<10 g/dL versus <8 g/dL]=0.427, *p* = 0.0040), spleen volume (HR [ > 2000 cm^3^ versus ≤ 2000 cm^3^]=1.905, *p* = 0.0159), and WBC (HR [ ≥ 10 versus <10 × 10^9^/L] = 4.498, *p* < 0.0001) were independent prognostic factors of OS after model selection (Table 2). In both studies, disease type (PMF, post-ET MF, or post-PV MF) was not predictive of OS.

**Table 1:** Univariate Cox regression analyses of baseline characteristics and overall survival (OS) in SIMPLIFY-1 and SIMPLIFY-2 study populations.

Subgroup	Number of Events/ Number of Evaluable Patients (%)	Median OS (years)	2-year OS (%)	Hazard Ratio (95% CI)	*p*-value
**SIMPLIFY-1**					
**TI**	**74/297 (24.9)**	**NR**	**86.0**	**0.373 (0.267, 0.523)**	**<0.0001**
non-TI	65/133 (48.9)	3.20	69.9	ref	
TSS ≥ 18	62/196 (31.6)	NR	78.9	1.059 (0.756, 1.482)	0.7408
TSS < 18	75/229 (32.8)	NR	82.6	ref	
**Hgb** ≥ **10** **g/dL**	**68/249 (27.3)**	**NR**	**83.3**	**0.351 (0.222, 0.557)**	**<0.0001**
**Hgb8-<10** **g/dL**	**45/131 (34.4)**	**5.01**	**86.1**	**0.493 (0.302, 0.805)**	
Hgb < 8 g/dL	25/49 (51.0)	3.76	55.6	ref	
**Spleen volume** > **2000** **cm**^**3**^	**74/208 (35.6)**	**NR**	**74.2**	**1.448 (1.037, 2.022)**	**0.0288**
Spleen volume ≤2000 cm^3^	65/221 (29.4)	NR	87.5	ref	
**Platelets** > **200** **×** **10**^**9**^**/L**	**73/260 (28.1)**	**NR**	**84.5**	**0.466 (0.281, 0.773)**	**0.0035**
**Platelets 100-200** **×** **10**^**9**^**/L**	**47/129 (36.4)**	**NR**	**78.0**	**0.713 (0.418, 1.215)**	
Platelets <100 × 10^9^/L	19/41 (46.3)	4.28	68.0	ref	
Disease type: PET	35/90 (38.9)	5.02	80.4	1.125 (0.755, 1.674)	0.2909
Disease type: PPV	25/96 (26.0)	NR	82.7	0.752 (0.480, 1.179)	
Disease type: PMF	79/244 (32.4)	NR	80.7	ref	
**IPSS risk: High**	**88/200 (44.0)**	**3.76**	**72.2**	**5.494 (2.920, 10.340)**	**<0.0001**
**IPSS risk: INT-2**	**40/143 (28.0)**	**NR**	**85.7**	**2.792 (1.430, 5.451)**	
IPSS risk: INT-1	11/87 (12.6)	NR	93.9	ref	
**WBC** ≥ **10** **×** **10**^**9**^**/L**	**79/219 (36.1)**	**5.31**	**76.4**	**1.466 (1.045, 2.056)**	**0.0258**
WBC < 10 × 10^9^/L	59/210 (28.1)	NR	86.0	ref	
**SIMPLIFY-2**					
**TI**	**12/51 (23.5)**	**NR**	**80.7**	**0.319 (0.171, 0.595)**	**0.0002**
non-TI	58/105 (55.2)	2.28	56.1	ref	
TSS ≥ 18	30/67 (44.8)	2.86	57.2	1.272 (0.792, 2.044)	0.3174
TSS < 18	40/89 (44.9)	5.31	69.3	ref	
**Hgb** ≥ **10** **g/dL**	**18/51 (35.3)**	**NR**	**67.9**	**0.335 (0.179, 0.624)**	**0.0003**
**Hgb 8-<10** **g/dL**	**29/72 (40.3)**	**5.31**	**71.7**	**0.493 (0.302, 0.805)**	
Hgb <8 g/dL	23/33 (69.7)	1.60	42.4	ref	
**Spleen volume** > **2000** **cm**^**3**^	**46/84 (54.8)**	**2.19**	**53.2**	**2.329 (1.418, 3.824)**	**0.0006**
Spleen volume ≤2000 cm^3^	24/72 (33.3)	NR	76.1	ref	
Platelets >200 × 10^9^/L	19/36 (52.8)	2.02	52.3	1.926 (1.063, 3.489)	0.0857
Platelets 100-200 × 10^9^/L	23/49 (46.9)	2.58	58.8	1.440 (0.821, 2.526)	
Platelets <100 × 10^9^/L	26/69 (37.7)	NR	76.1	ref	
Disease type: PET	12/32 (37.5)	NR	82.9	0.614 (0.326, 1.156)	0.0606
Disease type: PPV	9/30 (30.0)	NR	68.7	0.482 (0.237, 0.982)	
Disease type: PMF	49/94 (52.1)	2.76	56.4	ref	
**DIPSS risk: High**	**19/27 (70.4)**	**1.18**	**24.0**	**5.043 (2.374, 10.710)**	**<0.0001**
**DIPSS risk: INT-2**	**40/90 (44.4)**	**5.31**	**70.9**	**1.651 (0.847, 3.221)**	
DIPSS risk: INT-1	11/39 (28.2)	NR	73.9	ref	
**WBC** ≥ **10** **×** **109/L**	**37/59 (62.7)**	**1.33**	**38.4**	**3.335 (2.072, 5.365)**	**<0.0001**
WBC < 10 × 109/L	33/97 (34.0)	NR	78.6	ref	

*TI* Transfusion independence, *TSS* Total symptom score, *Hgb* Hemoglobin, *PET* Post-essential thrombocythemia, *PPV* Post-polycythemia vera, *PMF* Primary myelofibrosis; *IPSS*, International Prognostic Scoring System, *DIPSS* Dynamic International Prognostic Scoring System, *INT* Intermediate, *NR* Not reached, *Ref* Reference group, *WBC* White blood cells. Bold font highlights statistically significant associations.

**Table 2:** Multivariate Cox regression analyses of baseline characteristics and overall survival (OS) in SIMPLIFY-1 and SIMPLIFY-2 study populations.

Baseline Parameter	Hazard Ratio (95% CI)	*p*-value
**SIMPLIFY-1**		
IPSS risk: High vs INT-1	**4.293 (2.160, 8.532)**	**<0.0001**
IPSS risk: INT-2 vs INT-1	**2.759 (1.373, 5.546)**	**0.0044**
TI: Yes vs No	**0.474 (0.325, 0.691)**	**0.0001**
WBC: ≥ 10 vs <10 × 10^9^/L	**1.648 (1.160, 2.342)**	**0.0054**
**SIMPLIFY-2**		
TI: Yes vs No	**0.226 (0.097, 0.524)**	**0.0005**
Spleen volume > 2000 cm^3^: Yes vs No	**1.905 (1.128, 3.216)**	**0.0159**
Hgb: 8-<10 vs <8 g/dL	**0.427 (0.239, 0.762)**	**0.0040**
Hgb: ≥10 vs <8 g/dL	0.653 (0.294, 1.449)	0.2949
WBC: ≥ 10 vs <10 × 10^9^/L	**4.498 (2.655, 7.621)**	**<0.0001**

*IPSS* International Prognostic Scoring System, *INT* Intermediate, *TI* Transfusion independence, *WBC* White blood cells, *DIPSS* Dynamic International Prognostic Scoring System, *Hgb* hemoglobin, *CI* Confidence interval. Bold font highlights statistically significant associations.

**Fig. 2:**
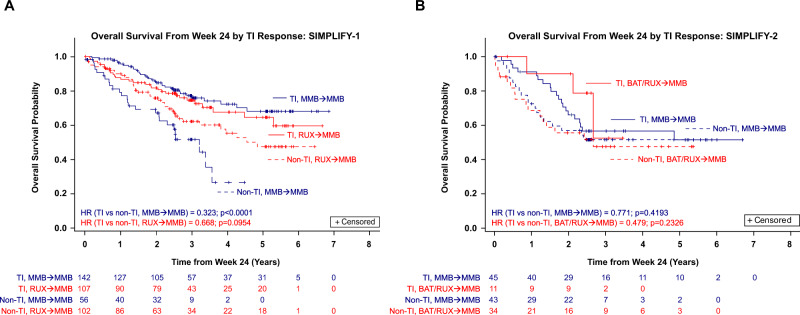
Association between transfusion independence (TI) response at week 24 and overall survival (OS) in patients with MF. **A** OS by TI response in JAKi-naïve patients in SIMPLIFY-1; (**B**) OS by TI response in JAKi-exposed patients in SIMPLIFY-2. *TI-NR* Transfusion independence non-responders, *TI-R* Transfusion independence responders.

In SIMPLIFY-1, TI response at week 24 in MMB-randomized patients was associated with improved OS, with a 3-year survival rate of 77.2% for TI responders (*N* = 142) compared to 51.6% for TI non-responders (*N* = 56) (HR = 0.323; *p* < 0.0001; Fig. 2A). Trends toward improvements in OS were observed in MMB-randomized TI responders at week 24 in SIMPLIFY-2, with a 2-year survival rate of 66.1% for TI responders (*N* = 45) compared to 57.0% for TI non-responders (*N* = 43) (HR = 0.771; *p* = 0.4193; Fig. 2B).

Trends toward improvements in OS were observed in week 24 TI responders randomized to RUX in SIMPLIFY-1 (HR = 0.668, *p* = 0.0954; Fig. 2A) and to BAT/RUX in SIMPLIFY-2 (HR = 0.479, *p* = 0.2326; Fig. 2B). Of the patients randomized to RUX followed by MMB in SIMPLIFY-1 who were non-TI at week 24 (*N* = 92), 42 (45.7%) became TI by week 36; of those who did not become TI by week 36 and for whom week 36 transfusion data was available (*N* = 37), 29 (78.4%) had decreased transfusion burden at week 36 compared to week 24.

**Fig. 3:**
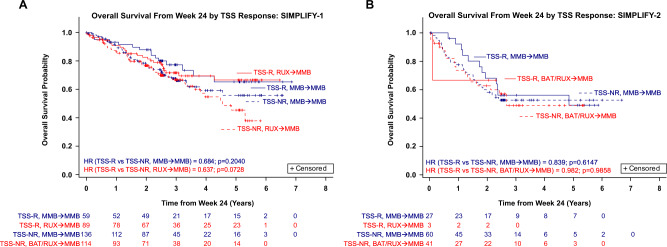
Association between total symptom score (TSS) response at week 24 and overall survival (OS) in patients with MF. **A** OS by TSS response in JAKi-naïve patients in SIMPLIFY-1; (**B**) OS by TSS response in JAKi-exposed patients in SIMPLIFY-2. *TSS-NR* total symptom score non-responders, *TSS-R* total symptom score responders.

**Fig. 4:**
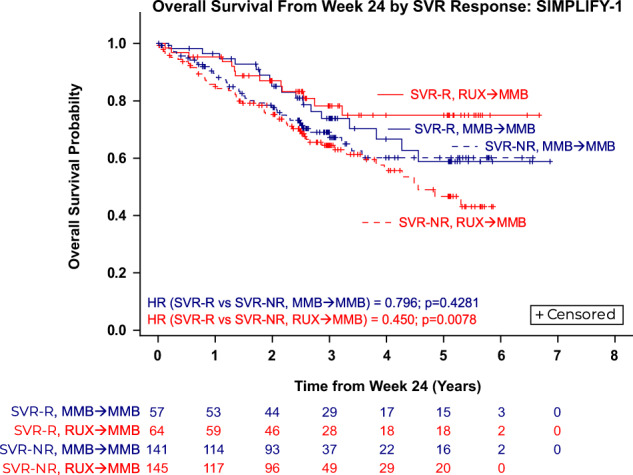
Association between spleen volume reduction (SVR) response at week 24 and overall survival (OS) in JAKi-naïve (SIMPLIFY-1) patients with MF. Due to the low number of splenic responders in SIMPLIFY-2 (MMB arm, *N* = 7/104 [7%]; BAT/RUX→MMB arm, *N* = 3/52 [6%]), the responder analysis was not interpretable for the SVR endpoint. *NR* Non-responders, *R* Responders.

Observations from SIMPLIFY-1 demonstrate a trend toward improved OS for MMB- and RUX-randomized patients who achieved a symptom response at week 24 versus non-responders (MMB HR = 0.684, *p* = 0.2040; RUX→MMB HR = 0.637, *p* = 0.0728; Fig. 3A). In SIMPLIFY-1, week 24 SVR response was associated with improved OS in RUX-randomized patients (RUX→MMB HR = 0.450, *p* = 0.0078), and a trend toward improved OS in MMB-randomized patients (MMB HR = 0.796, *p* = 0.4281; Fig. 4). In SIMPLIFY-2, no difference in OS was found between TSS responders and non-responders in either treatment arm (MMB HR = 0.839, *p* = 0.6147; BAT/RUX→MMB HR = 0.982, *p* = 0.9858; Fig. 3B).

Multivariate Cox regression analysis was performed to confirm the association between TI response at week 24 and OS in MMB-randomized patients in SIMPLIFY-1 in the presence of other response endpoints in the model. An independent association between MMB TI response at week 24 and OS in the SIMPLIFY-1 trial was found [HR = 0.311 (95% CI, 0.173-0.559), *p* < 0.0001]. Neither MMB splenic response [HR = 1.151 (95% CI, 0.613-2.161), *p* = 0.6613] nor MMB symptom response [HR = 0.853 (95% CI, 0.466-1.562), *p* = 0.6071] at week 24 was associated with OS in SIMPLIFY-1.

## Discussion

Here we present for the first time, mature survival data from the two phase 3 SIMPLIFY trials, which demonstrate that extended treatment with MMB is associated with excellent OS and LFS, regardless of whether a JAKi-naïve patient was initially randomized to MMB or to RUX followed by MMB in SIMPLIFY-1, or whether a previously RUX-treated patient was initially randomized to MMB or to BAT/RUX followed by MMB in SIMPLIFY-2. In the SIMPLIFY-1 non-inferiority study, the two treatment arms produced nearly identical OS and LFS outcomes, providing confidence that survival is similar for patients whose initial frontline JAKi is MMB or RUX. The median OS was not reached and the 5-year survival probability was ~55% in both arms of SIMPLIFY-1; while cross-study variability precludes a direct comparison, a 5-year survival rate of ~55% was also observed with RUX in COMFORT pooled analyses [13]. In the RUX-exposed setting of SIMPLIFY-2, patients were anemic and/or thrombocytopenic at study onset, and analogous historical clinical trial data for post-JAKi RUX is not available. Real-world studies estimate the median OS of patients who discontinue RUX to be 11–14 months, although there are no standardized criteria for RUX discontinuation [18, 19, 22, 29]. In a clinical trial setting, the median OS of ~3 years and the 2-year survival rate of ~61–66% observed in patients treated with extended MMB in SIMPLIFY-2 demonstrate excellent survival post-RUX.

Imparting MMB’s spectrum of clinical efficacy is its unique inhibition of not only JAK1 and JAK2, but also ACVR1/ALK2, a central player in iron homeostasis. MMB inhibition of ACVR1/ALK2 leads to decreased hepcidin – the master regulator of iron metabolism that is elevated in patients with MF – resulting in increased serum iron availability for erythropoiesis [26, 27]. Due to this distinctive mechanism of action, MMB has demonstrated considerable anemia benefit in patients with MF [27], including higher rates of week 24 TI compared with RUX, regardless of degree of baseline anemia, baseline platelet count, or transfusion status [23, 30]. As anemia and transfusion dependency are two of the leading adverse prognostic indicators in patients with MF [2–4], and elevated hepcidin is significantly associated with shortened OS in patients with MF [28], the durable survival outcomes observed with extended MMB treatment in the SIMPLIFY studies are potentially linked to MMB’s marked anemia and TI benefits. In addition, MMB’s favorable hematologic safety profile enables high dose intensity to be maintained over long treatment periods, potentially contributing to the durability of MMB benefits including long-term survival.

In the SIMPLIFY study populations, baseline TI status, Hgb levels, and other known prognostic baseline factors were predictive of OS, consistent with well-established clinical assessments. The new finding that TI response at week 24 in JAKi-naïve, MMB-randomized patients in SIMPLIFY-1 was significantly associated with improved OS may have clinically relevant implications for future treatment decision-making. A trend toward improved OS in TI responders was also observed in the JAKi-exposed SIMPLIFY-2 population, as well as in JAKi-naïve, RUX-randomized patients in SIMPLIFY-1, which may be partially attributed to a salvage effect provided by crossover to MMB at week 24. This is supported by an observed TI response by week 36 in nearly half of non-TI RUX-randomized patients who switched to MMB at week 24, and reduced transfusion burden by week 36 in over three-fourths of the remaining non-TI patients who switched from RUX to MMB at week 24. As of note, OS was not different between RUX-treated patients who were TI and not TI at week 24 in long-term pooled analyses from the COMFORT-I and COMFORT-II phase 3 trials [13]. However, transfusion need at several timepoints following treatment with RUX was associated with shortened survival in a real-world observational study in MF, and was subsequently incorporated into the Response to Ruxolitinib After 6 Months (RR6) prognostic model that may help to predict which patients will benefit from a prompt treatment switch from RUX [15].

Because MMB has demonstrated higher rates of TI compared with RUX, and because MMB may be given at full dosage for longer, MMB may be the optimal treatment choice to maximize survival in certain subsets of patients with MF. While future studies are needed to validate week 24 TI response as a potential surrogate endpoint reasonably likely to predict improved OS in patients receiving MMB, the likelihood of a patient achieving week 24 TI response may become an important consideration regarding the initial choice of treatment. Currently, spleen volume and symptoms are primary drivers of treatment selection, and guidelines place an emphasis on platelet counts as a critical clinical factor. Given the association found between TI response and OS in JAKi-naïve patients, anemia and the importance of managing anemia may become a more central consideration when evaluating treatment options, and MMB is uniquely poised to fill the critical gap of addressing anemia in MF patients.

There are limitations inherent to the week 24 crossover design of the SIMPLIFY trials, which may influence the OS findings from the SIMPLIFY-1 and SIMPLIFY-2 studies. Specifically, because of crossover, OS data is not directly comparable between MMB and control arms, and therefore the MMB treatment effect cannot not be accurately estimated. Both studies were designed to provide 24-week comparative data while the survival outcomes might be considered descriptive for patients treated with extended MMB as most control arm patients crossed over to MMB at a relatively early timepoint relative to the median survival follow-up of more than three years. Moreover, the design of SIMPLIFY-2 did not allow for a washout period of prior JAKi therapy, which may have influenced the specificity of MMB effects and impacted the non-significance of association between week 24 clinical endpoints and OS in this study. Future studies are needed to uncover potential factors beyond baseline hemoglobin and transfusion requirements that may predict which MMB-treated patients are likely to become TI responders versus TI non-responders.

Momelotinib’s anemia benefits are currently being further characterized in MOMENTUM (NCT04173494), a global phase 3 clinical trial in symptomatic and anemic patients previously treated with an approved JAKi, intended to support potential registration of momelotinib for the treatment of MF [31]. In addition to assessments of transfusion independence, symptoms, and splenomegaly, MOMENTUM will provide an opportunity to evaluate associations between MMB anemia benefit, transfusion burden and patient-reported measures of clinical benefit as well as survival.

## Supplementary information

Data Supplement

## Data Availability

Sierra Oncology commits to share clinical study data with qualified researchers to enable enhancement of public health. As such, Sierra will share anonymized patient-level data on request or if required by law or regulation. Qualified scientific and medical researchers can request patient-level data for studies of Sierra pharmaceutical substances listed on ClinicalTrials.gov and approved by health authorities in the United States and the EU. Patient-level data for studies of newly approved pharmaceutical substances or indications can be requested 9 months after US Food and Drug Administration and European Medicines Agency approvals. Such requests are assessed at Sierra’s discretion, and the decisions depend on the scientific merit of the proposed request, data availability, and the purpose of the proposal. If Sierra agrees to share clinical data for research purposes, the applicant is required to sign an agreement for data sharing before data release, to ensure that the patient data are de-identified. In case of any risk of re-identification on anonymized data despite measures to protect patient confidentiality, the data will not be shared. The patients’ informed consent will always be respected. If the anonymization process will provide futile data, Sierra will have the right to refuse the request. Sierra will provide access to patient-level clinical trial analysis datasets in a secured environment upon execution of the data sharing agreement. Sierra will also provide the protocol, statistical analysis plan, and the clinical study report synopsis if needed. For additional information or requests for access to Sierra clinical trial data for research purposes, please contact us at Medinfo@sierraoncology.com.
