# Supplementary material for: Overall survival in the SIMPLIFY-1 and SIMPLIFY-2 phase 3 trials of momelotinib in patients with myelofibrosis
Source: Leukemia. 2022 Jul 22;36(9):2261–8. doi: 10.1038/s41375-022-01637-7 (PMC9417985; doi:10.1038/s41375-022-01637-7)

**Data Supplement**

**Overall Survival in the SIMPLIFY-1 and SIMPLIFY-2 Phase 3 Trials of Momelotinib in Patients with Myelofibrosis**

Ruben Mesa^1^, Claire Harrison^2^, Stephen T. Oh^3^, Aaron T. Gerds^4^, Vikas Gupta^5^, John Catalano^6^, Francisco Cervantes^7^, Timothy Devos^8^, Marek Hus^9^, Jean-Jacques Kiladjian^10^, Ewa Lech-Maranda^11^, Donal McLornan^2^, Alessandro M. Vannucchi^12^, Uwe Platzbecker^13^, Mei Huang^14^, Bryan Strouse^14^, Barbara Klencke^14^, Srdan Verstovsek^15^

^1^UT Health San Antonio Cancer Center, San Antonio, TX; ^2^Guy's and St Thomas' NHS Foundation Trust, London, United Kingdom; ^3^Washington University School of Medicine, St. Louis, MO; ^4^Cleveland Clinic Taussig Cancer Institute, Cleveland, OH; ^5^Princess Margaret Cancer Centre, Toronto, Ontario; ^6^Monash University & Frankston Hospital, Frankston, Australia; ^7^Hospital Clinic, IDIBAPS, University of Barcelona, Barcelona, Spain; ^8^Department of Hematology, University Hospitals Leuven and Department of Microbiology and Immunology, Laboratory of Molecular Immunology (Rega Institute), KU Leuven, Leuven, Belgium; ^9^Medical University of Lublin, Lublin, Poland; ^10^Université de Paris, AP-HP, Hôpital Saint-Louis, Centre d’Investigations Cliniques, INSERM, CIC1427, Paris, France; ^11^Institute of Hematology and Transfusion Medicine, Warsaw, Poland; ^12^University of Florence and AOU Careggi, Florence, Italy; ^13^Leipzig University Hospital, Leipzig, Germany; ^14^Sierra Oncology Inc., San Mateo, CA; ^15^The University of Texas MD Anderson Cancer Center, Houston, TX

Supplemental Table 1. Most frequent Treatment Emergent Adverse Events (TEAE) by patient in (A) SIMPLIFY-1 and (B) SIMPLIFY-2. MMB, momelotinib; RUX, ruxolitinib; BAT, best available therapy.


| **SIMPLIFY-1** | **Randomization Treatment Period (Weeks 0-24)** | | **Extended Duration MMB Final Safety Analysis^c^ (N=411)** |
| --- | --- | --- | --- |
| **Patients with Grade 3/4 TEAE^a^, n (%)** | **MMB (N=214)** | **RUX (N=216)** |  |
| Any Grade 3/4 TEAE | 74 (34.6) | 94 (43.5) | 263 (64.0) |
| Thrombocytopenia | 15 (7.0) | 10 (4.6) | 60 (14.6) |
| Anemia | 13 (6.1) | 49 (22.7) | 54 (13.1) |
| Pneumonia | 5 (2.3) | 3 (1.4) | 32 (7.8) |
| **Patients with any Grade TEAE^b^, n (%)** |  |  |  |
| Any TEAE | 198 (92.5) | 206 (95.4) | 400 (97.3) |
| Anemia | 31 (14.5) | 81 (37.5) | 106 (25.8) |
| Diarrhea | 39 (18.2) | 43 (19.9) | 100 (24.3) |
| Thrombocytopenia | 40 (18.7) | 63 (29.2) | 99 (24.1) |
| Nausea | 34 (15.9) | 8 (3.7) | 86 (20.9) |
| Fatigue | 31 (14.5) | 26 (12.0) | 85 (20.7) |
| Headache | 38 (17.8) | 43 (19.9) | 68 (16.5) |
| Peripheral sensory neuropathy | 20 (9.3) | 12 (5.6) | 62 (15.1) |

^a^Grade 3/4 TEAE occurring in >5% patients in the Extended Duration MMB Final Safety Analysis population.

^b^TEAE occurring in >20% patients in the Extended Duration MMB Final Safety Analysis population plus headache and peripheral sensory neuropathy of any frequency.

^c^Final data cut in September 2021 with a median follow up of 19 months.

| **SIMPLIFY-2** | **Randomization Treatment Period (Weeks 0-24)** | | **Extended Duration MMB Final Safety Analysis^c^ (N=144)** |
| --- | --- | --- | --- |
| **Patients with Grade 3/4 TEAE^a^, n (%)** | **MMB (N=104)** | **BAT/RUX (N=52)** |  |
| Any Grade 3/4 TEAE | 57 (54.8) | 22 (42.3) | 105 (72.9) |
| Anemia | 14 (13.5) | 9 (17.3) | 34 (23.6) |
| Thrombocytopenia | 11 (10.6) | 3 (5.8) | 23 (16.0) |
| Asthenia | 5 (4.8) | 1 (1.9) | 11 (7.6) |
| Pneumonia | 2 (1.9) | 1 (1.9) | 10 (6.9) |
| Neutropenia | 5 (4.8) | 1 (1.9) | 9 (6.3) |
| **Patients with any Grade TEAE^b^, n (%)** |  |  |  |
| Any TEAE | 101 (97.1) | 46 (88.5) | 142 (98.6) |
| Diarrhea | 34 (32.7) | 8 (15.4) | 53 (36.8) |
| Cough | 18 (17.3) | 6 (11.5) | 39 (27.1) |
| Anemia | 16 (15.4) | 10 (19.2) | 38 (26.4) |
| Pyrexia | 15 (14.4) | 4 (7.7) | 37 (25.7) |
| Asthenia | 20 (19.2) | 11 (21.2) | 36 (25.0) |
| Thrombocytopenia | 18 (17.3) | 6 (11.5) | 35 (24.3) |
| Nausea | 20 (19.2) | 5 (9.6) | 33 (22.9) |
| Fatigue | 16 (15.4%) | 10 (19.2%) | 30 (20.8) |
| Headache | 16 (15.4) | 3 (5.8) | 25 (17.4) |
| Peripheral sensory neuropathy | 8 (7.7) | 0 | 20 (13.9) |

^a^Grade 3/4 TEAE occurring in >5% patients in the Extended Duration MMB Final Safety Analysis population.

^b^TEAE occurring in >20% patients in the Extended Duration MMB Final Safety Analysis population plus headache and peripheral sensory neuropathy of any frequency.

^c^Final data cut in September 2021 with a median follow up of 10 months.

Supplemental Figure 1. High MMB dose intensity was maintained throughout the 24-week study periods and beyond in both SIMPLIFY-1 (A) and SIMPLIFY-2 (C) trials, whereas attenuated starting doses and progressive dose reductions of RUX were required due to induced or exacerbated myelosuppression in SIMPLIFY-1 (B) and SIMPLIFY-2 (D).


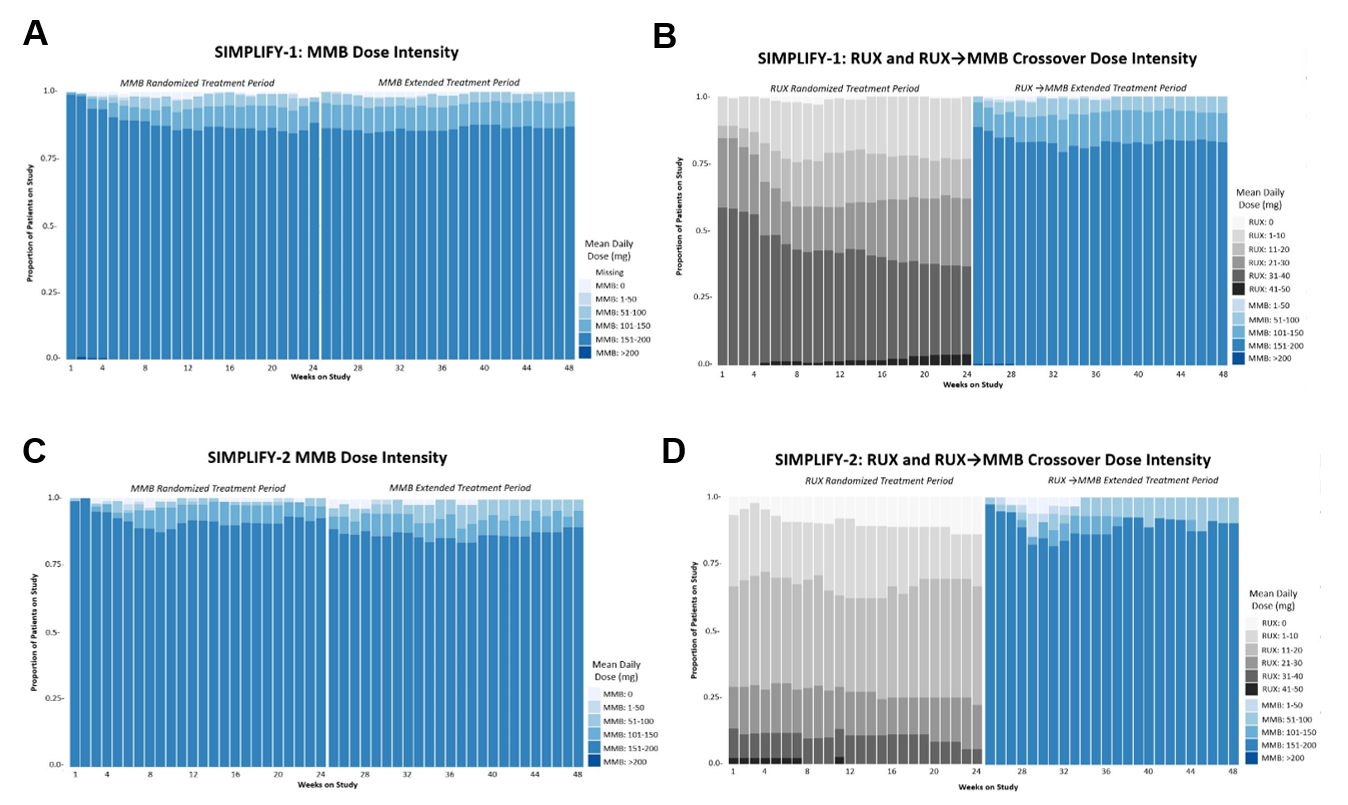

Supplement: Supplementary file 1 — Data Supplement [file 41375_2022_1637_MOESM1_ESM.docx]
